# Genetic Characterisation of Colistin Resistant *Klebsiella pneumoniae* Clinical Isolates From North India

**DOI:** 10.3389/fcimb.2021.666030

**Published:** 2021-06-21

**Authors:** Sanjay Singh, Ashutosh Pathak, Mohibur Rahman, Avinash Singh, Soumyabrata Nag, Chinmoy Sahu, Kashi Nath Prasad

**Affiliations:** ^1^ Department of Microbiology, Sanjay Gandhi Post Graduate Institute of Medical Sciences, Lucknow, India; ^2^ Department of Microbiology, Apollomedics Super Speciality Hospital, Lucknow, India

**Keywords:** bla_NDM_, colistin resistance, Klebsiella pneumoniae, mcr-1, mgrB, sequence type

## Abstract

**Background:**

Increasing use of colistin has led to the world-wide emergence of mobile colistin resistant gene (*mcr*). The present study aimed to identify and characterise mcr and other drug-resistant genes in colistin resistant *Klebsiella pneumoniae* clinical isolates.

**Methods:**

Twenty-two colistin resistant *K. pneumoniae* were analysed for mcr and other drug-resistant genes, efflux pumps, and virulence genes, and for their biofilm forming ability. Pulsed-field gel electrophoresis (PFGE) and multi-locus sequence typing (MLST) were performed for all *mcr-1* positive isolates. S1-PFGE and Southern hybridisation were performed for localisation of *mcr-1* and *bla*_NDM_.

**Results:**

Nineteen colistin resistant *K. pneumoniae* harboured *mcr-1* and 3 had *mgrB* disruption. All isolates harboured *bla*_OXA-48_-type and ESBL genes; eight strains (five with *mcr-1* and three with *mgrB* disruption) co-harboured *bla*_NDM_. Efflux pumps genes *AcrAB* and *mdtK* were detected in all 22 and *tol-C* in 21 isolates. Virulence-related genes entB and *irp-1* were detected in all 22, *mrkD* in 20, and *fimH-1* in 18 isolates; 11 isolates were strong biofilm producers. PFGE clustered *mcr-1* positive isolates into eight groups based on ≥90% similarity; MLST revealed diverse sequence types, predominant being ST-15 (n = 4) and ST-16 (n = 4). Both *mcr-1* and *bla*_NDM_ were localised on plasmid and chromosome; *mcr-1* was present on IncFII type and *bla*_NDM_ on IncFIB and IncA/C type plasmids.

**Conclusions:**

Colistin resistance in *K. pneumoniae* was predominantly mediated by *mcr-1*. Co-existence of colistin, carbapenem, and other drug-resistant genes along with efflux pumps indicates towards enormous genomic plasticity in *K. pneumoniae* with ability to emerge as super-spreader of drug-resistance.

## Introduction

Increasing prevalence of multi-drug resistant (MDR) Gram-negative bacteria (GNB) is a serious public health concern since they are susceptible only to few antibiotics (Laws et al., 2019). The World Health Organization (WHO) has listed carbapenem-resistant *Klebsiella pneumoniae* among the priority pathogen group as it poses great threat to human health (WHO, 2017). *K. pneumoniae* belongs to the Enterobacteriaceae family and is a common nosocomial pathogen responsible for significant morbidity and mortality. Virulence factors such as capsular polysaccharides, lipopolysaccharide (LPS), siderophores and adherence factors help *K. pneumoniae* to circumvent host immune response and increase its pathogenicity. Biofilm formation also plays a significant role in drug resistance and inflammation resulting in persistent infections (Navon-Venezia et al., 2017).

Colistin is the last resort drug of choice for treatment of lethal infections caused by carbapenem resistant GNB. Colistin is a cationic polypeptide antibiotic that binds to the negatively charged phosphate group of LPS of GNB, which results in disarrangement of cell membrane. Ultimately, there is a loss of cell membrane integrity resulting in increased permeability of the cell, leakage of cell contents, and finally cell lysis (Baron et al., 2016). The re-introduction of colistin in clinical practice has resulted in its increased reports of resistance in GNB. Resistance to colistin is either chromosomal or plasmid mediated. Mobile colistin resistant gene (*mcr-1*) located either on chromosome or on plasmid encodes phosphoethanolamine transferase. Since the first report of *mcr-1* in late 2015, ten different *mcr* variants (*mcr-1* to *mcr-10*) have been reported (Wang et al., 2020).

In this study, we investigated the presence of *mcr* in colistin resistant *K. pneumoniae* strains. Such strains were also examined for the presence of other drug-resistant genes and also for virulence and efflux pumps genes and for their ability to form biofilm. Analyses of clonal relatedness and strain typing were performed in *mcr-1* positive isolates. Further, characterisation of plasmids harbouring both *mcr-1* and *bla*_NDM_ was also performed.

## Materials and Methods

### Bacterial Strains

The study was conducted at Sanjay Gandhi Postgraduate Institute of Medical Sciences (Lucknow, India), a 900 bed tertiary care referral hospital in North India. Twenty-two colistin resistant *K. pneumoniae* isolates recovered from various clinical samples like pus, blood, endotracheal aspirate, tissue, and sputum between October 2016 and March 2017 were included in the study. All the isolates were identified using biochemical tests and MALDI-TOF MS (BioMérieux, Marcy l’Étoile, France). Prior to testing, all the isolates were stored in brain heart infusion broth (Becton, Dickinson and Company, Sparks, USA) supplemented with 20% glycerol (Sigma-Aldrich, MO, USA) at −80°C.

### Demographic and Clinical Data

Demographic and clinical data of patients were obtained from the hospital information system available in the hospital intranet.

### Antimicrobial Susceptibility Testing

Minimum inhibitory concentrations (MICs) were determined by broth microdilution method (BMD) in cation adjusted Mueller–Hinton broth following Clinical and Laboratory Standards Institute (CLSI) guidelines except colistin for which European Committee on Antimicrobial Susceptibility Testing breakpoints were followed (CLSI, 2017; EUCAST, 2017). Isolates were considered MDR if they were resistant to at least one antibiotic of three different classes among those tested (cephalosporins, carbapenems, fluoroquinolones, aminoglycosides, and polymyxins) according to Magiorakos et al. (2012).

### DNA Isolation, Detection of Antibiotic Resistance, Efflux Pump and Virulence Genes

DNA was extracted from overnight grown culture using Wizard Genomic DNA Purification Kit (Promega, WI, USA). Genomic DNA quality was measured by NanoDrop ND-2000 spectrophotometer (Thermo Fisher Scientific, DE, USA). The integrity of genomic DNA was analysed by agarose gel electrophoresis. The extracted DNA was stored at −20°C.

The presence of *mcr* genes (*mcr-1* to *mcr-8*) was analysed by conventional PCR, and the amplified products were confirmed by sequencing. The *mcr* positive isolates were also examined for the presence of carbapenemases (*bla*_IMP_, *bla*_KPC_, *bla*_NDM_, *bla*_VIM_, and *bla*_OXA-48_ type), extended spectrum *β*-lactamases (ESBLs; *bla*_TEM_, *bla*_SHV_, and *bla*_CTX-M_), 16S rRNA methyltransferases (*armA* and *rmtA-F*). List of primers is given in **Table S1**.

Chromosomal mutations were analysed in isolates negative for *mcr*. Conventional PCR was performed using specific primers (**Table S1**) to detect mutations in *mgrB, phoP/phoQ, pmrA, and pmrB.* The PCR products were purified, Sanger sequenced, and analysed to determine the mutations responsible for colistin resistance.

K. pneumoniae strains positive for mcr were also screened by conventional PCR for the presence of genes encoding for multidrug efflux pump systems like ArcAB, TolC, and MdtK, and virulence determinants such as regulator of mucoid phenotype (*rmpA*), type 1 and type 3 adhesins (*fimH-1* and *mrkD*), iron siderophores (aerobactin synthase, *luc*C), bacteriocin biosynthesis [enterobactin (entB), and yersiniabactin (irP-1)], and serum resistance-associated outer membrane lipoprotein (traT).

### Capsular Typing

Capsular typing based on wzi gene sequence was done as reported previously (Brisse et al., 2013). The PCR products were Sanger sequenced, and wzi alleles were identified, and corresponding capsular polysaccharide types (KL-types) were determined by comparing our *wzi* sequences with those available on the *Klebsiella* PasteurMLST sequence definition database (https://bigsdb.pasteur.fr/).

### Biofilm Assay

Biofilm assay was performed by O’Toole and Kolter’s protocol with little modification (O’toole and Kolter, 1998). Briefly, 1 µl of overnight grown culture was inoculated into 100 µl of fresh tryptone soya broth (TSB) in 96 well sterile flat bottom polystyrene plates. After overnight incubation at 37°C, the cultures in wells were discarded. The wells were washed gently with water followed by air drying for 15 min. Biomass was stained with 125 µl of 0.1% (w/v) crystal violet for 20 min. Plates were rinsed off, air dried, and the dye bound to adherent biomass was eluted with 30% acetic acid. Absorbance was measured using automated microplate reader (MultiskanGO, Thermo Scientific, MA, USA) at 570 nm. Tests were performed in triplicate, and results were averaged. The results were interpreted according to Stepanovic et al. (2000). *K. pneumoniae* ATCC strain, ATCC 700603 was used as positive control whereas *E. coli* K-12 was used as negative control.

### Clonal Diversity and Strain Typing

Clonal diversity among 19 mcr-1 positive K. pneumoniae isolates was examined by pulsed field gel electrophoresis (PFGE) according to previously reported protocol (Qin et al., 2014). Banding patterns were analysed using BioNumerics software version 7.6 (Applied-Maths, Sint-Martens-Latem, Belgium). *Salmonella* serotype Branderup strain (H9812) digested with *XbaI* (Promega, WI, USA) was used as reference strain.

Multi-locus sequence type (MLST) of 19 *mcr-1* positive *K. pneumoniae* isolates was analysed as described previously (Diancourt et al., 2005). The seven housekeeping genes were amplified and sequenced. The sequence type (ST) was assigned by determining the allele number for each of the housekeeping genes using the database maintained by Pasteur Institute at http://bigsdb.pasteur.fr/klebsiella/klebsiella.html/.

### Conjugation Experiment and Plasmid Replicon Typing

Horizontal gene transfer ability of *bla*_NDM_ and *mcr-1* was determined using liquid mating assay for five *K. pneumoniae* isolates that harboured both *mcr-1* and *bla*_NDM_. *E. coli* J53 was used as recipient strain, and transconjugants selection was performed on MacConkey agar plates containing meropenem (2 µg/ml) or colistin (1.0 µg/ml) as applicable and sodium azide (100 µg/ml). Transconjugants were tested for *mcr-1* or *bla*_NDM_ by PCR and antimicrobial susceptibility. Plasmid DNA was isolated from transconjugants following Kado and Liu method (Kado and Liu, 1981). PCR-based replicon typing (PBRT) was done to determine the plasmid incompatibility group (Carattoli et al., 2005).

### S1-PFGE and Hybridisation

S1 PFGE and Southern hybridisation were performed for five strains that harboured both *bla*_NDM_ and *mcr-1*. Bacterial DNA was prepared in agarose plugs, digested with S1 nuclease (Promega, WI, USA), and the linearised plasmid was then separated through PFGE. The gel was stained with ethidium bromide and transferred to nylon membrane (Hybond N, Amersham, UK) followed by hybridisation with digoxigenin labelled probes specific to *mcr-1* or *bla*_NDM_. Probe labelling and signal detection were done by DIG DNA Labeling and Detection Kit (Roche Diagnostics, GmbH, Germany).

## Results

### Bacterial Isolates and Patient Details

Twenty-two colistin resistant *K. pneumoniae* isolates recovered from 22 (male 18) patients were analysed; 12 patients were from post-operative intensive care unit (ICU) and four from critical care medicine, three from nephrology, two from paediatric gastroenterology, and one from haematology wards. Most of the isolates were recovered from endotracheal aspirate (45.4%, 10/22), followed by blood (27.3%, 6/22) and sputum (9.1%, 2/22). All isolates except one were recovered after 48 h of admission. Among the 12 post-operative ICU patients, 66.7% (8/12) succumbed to their infection. Co-morbidities were present in 86.4% (19/22) of patients. Hypertension was present in 36.4% (8/22), followed by acute kidney injury (13.6%, 3/22), type-2 diabetes, and chronic liver disease in 9.1% (2/22) each. The clinical details of all patients are given in **Table 1**.

**Table 1:** Demographic and clinical features of patients infected with colistin resistant *Klebsiella pneumoniae*.

Isolate ID	Sex/Age	Specimen	Days from admission to isolation of CR*kp*	Ward	Diagnosis	Type of infection	Co-morbidity	Outcome
CR*kp*1	M/40	Intra-abdominal fluid	26	Critical Care Medicine (CCM)	Alcohol pancreatitis	Intra-abdominal	Alcoholic, smoker, recurrent pancreatitis	Recovered and discharged
CR*kp*2	M/10	Tissue	8	Pediatric short stay unit	Wilm’s tumour on chemotherapy	Gangrene in left leg	Nil	Left leg amputation, recovered and discharged
CR*kp*3	M/82	Endotracheal (ET) aspirate	31	Medical-ICU	Septic shock, LRTI	LRTI	Hypertension, CAD, AMI	Recovered and discharged
CR*kp*4	M/36	ET aspirate	9	Post-operative ICU	CLD with bilateral pneumonia and septic shock	LRTI	Nil	Death
CR*kp*5	M/61	ET aspirate	3	CCM	Acute febrile illness	LRTI	AKI, Acute liver failure	Death
CR*kp*6	M/72	Femoral catheter tip	18	Nephrology ward	Septic shock, renal failure	Infected catheter tip	Hypertension	Death
CR*kp*7	F/36	Sputum	5	Post-operative ICU	Acute severe pancreatitis	Pneumonia	T2DM	Recovered and discharged
CR*kp*8	F/41	ET aspirate	25	Nephrology Ward	CKD, LRTI, septic shock	LRTI	Hypertension, Anemia, CKD	Death
CR*kp*9	F/26	ET aspirate	28	Nephrology ICU	Post-partum AKI, MODS, septic shock	LRTI	AKI, Anaemia	Death
CR*kp*10	M/34	Purulent discharge from left calf	39	Haematology ward	ALL (B cell)	Soft tissue infection/abscess	Nil	Recovered and discharged
CR*kp*11	M/58	ET aspirate	30	Post-operative ICU	Gunshot injury (face), LRTI, pyogenic meningitis, septic shock	LRTI	Multiple myeloma	Death
CR*kp*12	F/53	ET aspirate	14	Post-operative ICU	MODS, septic shock	LRTI	RHD (MS), PAH, CVA	Death
CR*kp*13	M/2	Blood	23	Paediatric gastroenterology ward	Septic shock	CLABSI	Neonatal cholestasis, enterocholitis	Recovered and discharged
CR*kp*14	M/59	ET aspirate	3	Nephrology ward	Septic shock, LRTI	LRTI	Hypertension, T2DM, CKD	Death
CR*kp*15	M/39	Blood	18	Post-operative ICU	Severe acute pancreatitis, intra-abdominal sepsis, multi-organ failure	Blood stream infection	Hypertension, alcoholic	Death
CR*kp*16	M/30	Blood	10	Pulmonary medicine ICU	Septic shock	Blood stream infection	Alcoholic liver disease, disseminated TB	Recovered and discharged
CR*kp*17	M/36	Blood	14	Post-operative ICU	Pneumonitis, septic shock	Blood stream infection	CLD	Death
CR*kp*18	M/53	Blood	8	Post-operative ICU	Hepatic encephalopathy, MODS	Blood stream infection	CLD	Death
CR*kp*19	M/82	Sputum	35	Medical -ICU	Septic shock, LRTI	LRTI	Hypertension, CAD, AMI	Recovered and discharged
CR*kp*20	M/70	ET aspirate	1	CCM	Systemic hypertension, COPD with type 1 respiratory failure	Ventilator associated pneumonia	Systemic hypertension	Recovered and discharged
CR*kp*21	M/19	ET aspirate	3	CCM	Severe acute pancreatitis	Ventilator associated pneumonia	Non-oliguric AKI	Recovered and discharged
CR*kp*22	M/55	Blood	11	Post-operative ICU	Gastric Carcinoma	septic shock	Hypertension	Death

ALL, Acute lymphocytic leukemia; AKI, Acute Kidney Injury; AMI, Acute myocardial infarction; CKD, Chronic kidney disease; CLABSI, Central line associated blood stream infection; CLD, Chronic liver disease; CVA, Cerebrovascular accident; ICU, Intensive Care Unit; LRTI, Lower respiratory tract infection; MODS, Multi-organ dysfunction syndrome; PAH, Pulmonary arterial hypertension; RHD (MS), Rheumatic heart disease (mitral stenosis); T2DM, Type 2 diabetes mellitus.

### Antimicrobial Susceptibility Testing

The antimicrobial susceptibility profile showed that all the isolates were MDR as they were non-susceptible to at least one antibiotic from three or more antibiotics classes. All 22 isolates were resistant to carbapenems (imipenem and meropenem), 3^rd^ generation cephalosporins (ceftazidime and ceftriaxone), monobactam (aztreonam), aminoglycoside (gentamicin), and fluoroquinolones (ciprofloxacin). The MIC values for colistin ranged from 8 to ≥512 mg/L. The antibiotic susceptibility results of 22 isolates are summarised in **Table 2**.

**Table 2:** Antimicrobial susceptibility profile and molecular characterisation of 22 colistin resistant *Klebsiella pneumonia*.

Isolate	MIC (mg/L)								Mechanism of colistin resistance	Resistance genes	Virulence genes	Genes coding for efflux pumps	Capsular type	Biofilm forming capacity
	IMI	MEM	CT	CAZ	CRO	AZT	GEN	CIP						
CR*kp*1	8	16	≥512	≥512	≥512	≥512	≥512	512	*mcr-1*	*bla_OXA-48_, bla_CTX-M_, bla_TEM_, bla_SHV_, bla_VIM_*	*mrkD, FimH-1, Ent B, Irp-1*,	*mdtK, tol-C, Acr-AB*	KL155	Strongly adherent
CR*kp*2	8	8	8	≥512	≥512	≥512	≥512	512	*mcr-1*	*bla_OXA-48_, bla_CTX-M_, bla_TEM_, bla_SHV_, bla_VIM_*	*mrkD, FimH-1, Ent B, Irp-1, traT*	*mdtK, Acr-AB*	KL112	Weakly adherent
CR*kp*3	8	16	16	≥512	≥512	256	128	≥512	*mcr-1*	*bla_OXA-48_, bla_CTX-M_, bla_TEM_, bla_SHV_, bla_VIM_*	*Ent B, Irp-1*	*mdtK, tol-C, Acr-AB*	KL51	Moderately adherent
CR*kp*4	256	128	≥ 512	≥512	≥512	≥512	≥512	512	*mcr-1*	*bla_OXA-48_, bla_CTX-M_, bla_TEM_, bla_SHV_, bla_VIM_*	*mrkD, FimH-1, Ent B, Irp-1*	*mdtK, tol-C, Acr-AB*	KL10	Strongly adherent
CR*kp*5	16	32	8	≥512	≥512	128	512	128	*mcr-1*	*bla_OXA-48_, bla_CTX-M_, bla_TEM_, bla_SHV_*	*mrkD, FimH-1, Ent B, Irp-1*	*mdtK, tol-C, Acr-AB*	KL155	Moderately adherent
CR*kp*6	4	8	8	≥512	≥512	≥512	≥512	512	*mcr-1*	*bla_OXA-48_, bla_CTX-M_, bla_TEM_, bla_SHV_, bla_VIM_*	*mrkD, FimH-1, Ent B, Irp-1, traT*	*mdtK, tol-C, Acr-AB*	KL30	Strongly adherent
CR*kp*7	8	8	16	512	128	32	4	16	*mcr-1*	*bla_OXA-48_, bla_VIM_*	*mrkD, FimH-1, Ent B, Irp-1*	*mdtK, tol-C, Acr-AB*	KL2	Moderately adherent
CR*kp*8	32	16	256	≥512	≥512	≥512	≥512	512	*mcr-1*	*bla_OXA-48_, bla_CTX-M_, bla_SHV_, bla_VIM_*	*mrkD, FimH-1, Ent B, Irp-1, traT*	*mdtK, tol-C, Acr-AB*	KL15	Strongly adherent
CR*kp*9	16	32	32	256	≥512	≥512	128	128	*mcr-1*	*bla_OXA-48_,bla_SHV_, bla_VIM_*	*mrkD, FimH-1, Ent B, Irp-1, traT*	*mdtK, tol-C, Acr-AB*	KL30	Strongly adherent
CR*kp*10	32	32	8	≥512	256	128	256	256	*mcr-1*	*bla_OXA-48_,bla_CTX-M_, bla_SHV_, bla_VIM_*	*mrkD, FimH-1, Ent B, Irp-1*	*mdtK, tol-C, Acr-AB*	KL149	Moderately adherent
CR*kp*11	32	64	256	≥512	≥512	≥512	≥512	512	*mcr-1*	*bla_OXA-48_, bla_NDM_, amp-c, bla_CTX-M,_ rmtC*	*rmpA, Ent B, Irp-1, traT*	*mdtK, tol-C, Acr-AB*	KL10	Strongly adherent
CR*kp*12	32	64	32	≥512	≥512	512	512	512	*mcr-1*	*bla_OXA-48_, bla_NDM,_ amp-c, bla_CTX-M_, bla_SHV_, rmtC*	*rmpA, mrkD, Ent B, FimH-1, Irp-1*	*mdtK, tol-C, Acr-AB*	KL18	Strongly adherent
CR*kp*13	256	256	128	≥512	≥512	≥512	≥512	512	*mcr-1*	*bla_OXA-48_, bla_NDM_, bla_TEM_, bla_CTX-M_, bla_SHV_, rmtB*	*mrkD, FimH-1, Ent B, Irp-1*	*mdtK, tol-C, Acr-AB*	KL155	Strongly adherent
CR*kp*14	8	16	16	≥512	≥512	≥512	128	256	*mcr-1*	*bla_OXA-48_, bla_VIM_, bla_CTX-M_, bla_TEM_, bla_SHV_*	*mrkD, FimH-1, Ent B, Irp-1*	*mdtK, tol-C, Acr-AB*	KL2	Weakly adherent
CR*kp*15	32	64	16	≥512	≥512	≥512	≥512	512	*mcr-1*	*bla_OXA-48_,bla_VIM_, bla_CTX-M_, bla_TEM_, bla_SHV_*	*mrkD, Ent B, Irp-1, traT*	*mdtK, tol-C, Acr-AB*	KL149	Moderately adherent
CR*kp*16	32	16	64	≥512	≥512	≥512	≥512	512	*mcr-1*	*bla_OXA-48_,bla_CTX-M_, bla_TEM_, bla_SHV_*	*mrkD, FimH-1, Ent B, Irp-1*	*mdtK, tol-C, Acr-AB*	KL51	Weakly adherent
CR*kp*17	64	128	32	≥512	≥512	≥512	≥512	512	*mcr-1*	*bla_OXA-48_, bla_NDM_, bla_CTX-M_, bla_TEM_, bla_SHV_, armA*	*mrkD, FimH-1, Ent B, Irp-1*	*mdtK, tol-C, Acr-AB*	KL10	Strongly adherent
CR*kp*18	32	16	64	≥512	≥512	≥512	≥512	512	*mcr-1*	*bla_OXA-48_, bla_NDM_, bla_CTX-M_, bla_SHV_, bla_TEM_*	*mrkD, FimH-1, Ent B, Irp-1*	*mdtK, tol-C, Acr-AB*	KL30	Strongly adherent
CR*kp*19	4	8	64	≥512	≥512	≥512	≥512	512	*mcr-1*	*bla_OXA-48_, bla_CTX-M_, bla_TEM_, bla_SHV_*	*Ent B, Irp-1*	*mdtK, tol-C, Acr-AB*	KL2	Moderately adherent
CR*kp*20	≥512	8	16	64	64	64	128	32	*mgrB*	*bla_OXA-48_, bla_IMP_, bla_NDM_, bla_SHV_, bla_CTX-M_, armA*	*mrkD, FimH-1, Ent B, Irp-1*	*mdtK, tol-C, Acr-AB*	KL18	Moderately adherent
CR*kp*21	≥512	32	8	64	128	256	256	128	*mgrB*	*bla_OXA-48_, bla_VIM_, bla_NDM_, bla_SHV_, bla_TEM_, bla_CTX-M_, armA*	*mrkD, Ent B, Irp-1*	*mdtK, tol-C, Acr-AB*	KL30	Strongly adherent
CR*kp*22	512	128	4	64	128	128	4	8	*mgrB*	*bla_OXA-48_, bla_VIM_, bla_NDM_, bla_SHV_, bla_TEM_, bla_CTX-M_, armA*	*mrkD, Ent B, Irp-1*	*mdtK, tol-C, Acr-AB*	KL10	Moderately adherent

CT, Colistin; IMI, Imipenem; MEM, Meropenem; CAZ, Ceftazidime; CRO, Ceftriaxone; AZT, Aztreonam; GEN, Gentamicin; CIP, Ciprofloxacin.

### PCR Based Detection of Resistant Genes

Nineteen (86.4%) of 22 colistin resistant isolates harboured *mcr-1*, and the remaining three (13.6%) had *mgrB* disruption. Eight (36.4%) strains harboured *bla*_NDM_; five and three of them were positive for *mcr-1* and *mgrB* disruption respectively. All 22 isolates carried *bla*_OXA-48_-type gene; *bla*_VIM_ was detected in 13 (59.0%) isolates and *bla*_IMP_ in one (4.5%) isolate. Twenty (90.1%) isolates harboured both *bla*_CTX-M_ and *bla*_SHV_, whereas *bla*_TEM_ was detected in 15 (68.2%) isolates. 16S r-RNA methyl transferase was detected in seven (31.8%) isolates (*armA* in four, *rmtB* in one, and *rmtC* in two isolates). Distributions of resistance genes in different combinations are given in **Table 2**.

PCR amplification of *mgrB* in three *K. pneumoniae* isolates (CR*kp*20, CR*kp*21, and CR*kp*22) revealed a larger (~1000 bp) amplicon. Sequencing of the amplicons showed *IS* elements mediated *mgrB* disruption. The *IS* elements involved in *mgrB* disruption belonged to *IS*1-like (777 bp) in CR*kp*20 and *IS*5-like families, 1,066 bp and 1,196 bp in CR*kp*21 and CR*kp*22 respectively. None of the isolates had mutation in *phoP/phoQ, pmrA, and pmrB*gene.

### Detection of Efflux Pump and Virulence Genes

All 22 colistin resistant strains harboured *AcrAB*, *mdtK*, and *tol-C* efflux pumps except one isolate that lacked *tol-C* (**Table 2**). Mucoid phenotype regulator, *rmpA*, was identified in two isolates. The siderophore associated genes, *entB* and *irp-1*, were present in all the isolates. Other virulence genes *fimH-1*, *mrkD*, and *traT* were detected in 16 (72.7%), 19 (86.4%), and six (27.3%) isolates respectively. The distribution of virulence genes is shown in **Table 2**.

### Biofilm Forming Capacity

*In-vitro* biofilm forming ability assay indicated that all 22 isolates were biofilm producers; 11 (50%) were strong, eight (36.4%) were moderate, and three (13.6%) were weak biofilm producers (**Table 2**).

### Capsular Typing

Wzi based capsular typing of colistin resistant *K. pneumoniae* indicated a high diversity as it predicted 10 different capsular polysaccharide serotypes (KL155 (n = 3), KL112 (n = 1), KL51 (n = 2), KL10 (n = 4), KL30 (n = 2), KL2 (n = 3), KL15 (n = 1), KL30 (n = 2), KL149 (n = 2), KL18 (n = 2).

### Clonal Diversity and Molecular Typing

**Figure 1:**
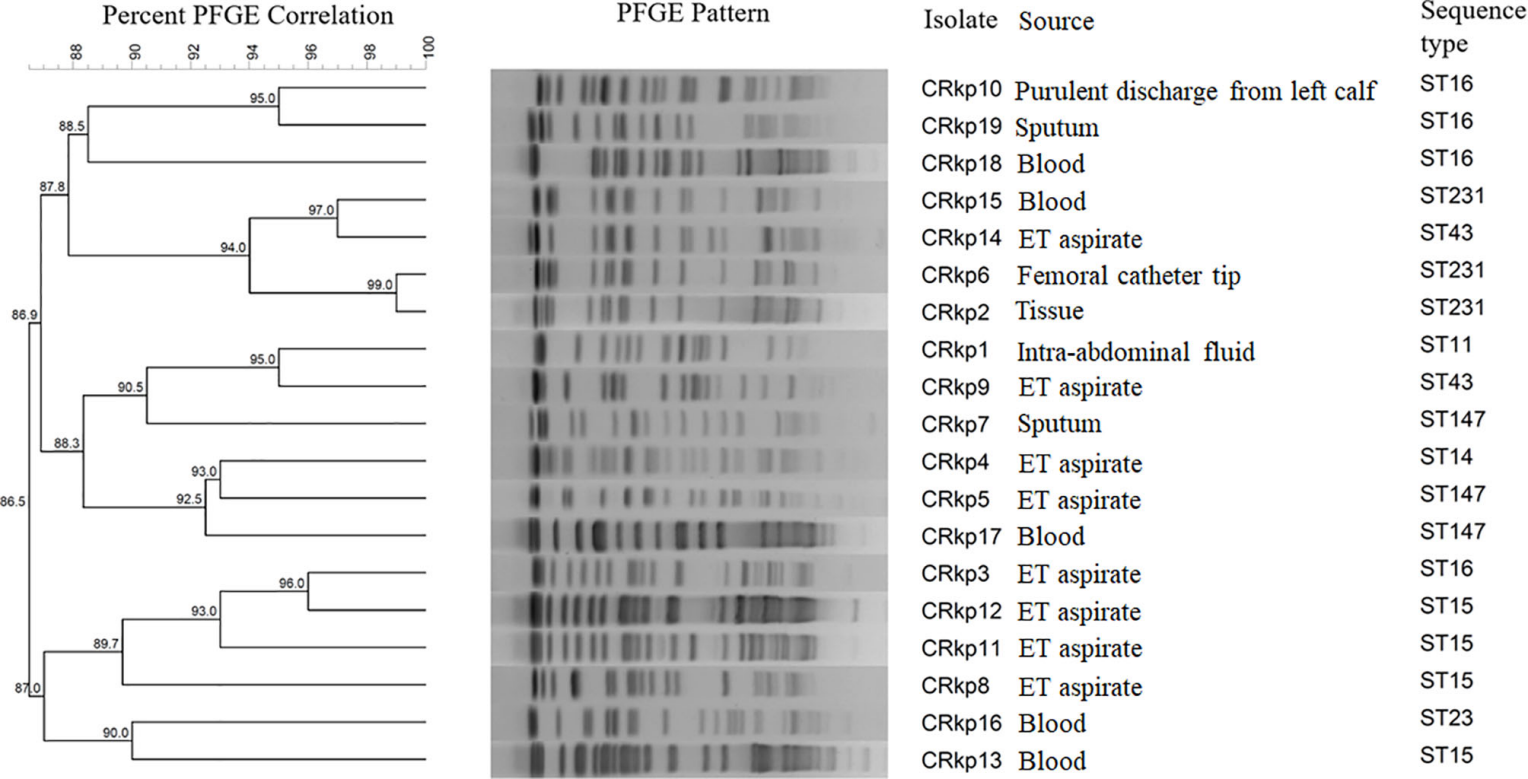
A dendrogram of the pulsed-field gel electrophoresis (PFGE) fingerprinting results and sequence types of 19 *mcr-1* positive *Klebsiella pneumoniae*.

All 19 *mcr-1* positive *K. pneumoniae* isolates were typeable by PFGE. The maximum and minimum genetic similarity observed between the isolates was 99 and 86.5% respectively (**Figure 1**). Based on ≥90% similarity they were clustered into eight groups.

MLST analysis of 19 *mcr-1* positive *K. pneumoniae* revealed eight different STs and their distributions were as follows: ST-15 (n = 4), ST-16 (n = 4), ST-231 (n = 3), and ST-147 (n = 3), ST-43 (n = 2) and one isolate each for ST-14, ST-11, and ST-23. The source of strains and their STs are shown in **Figure 1**.

### Conjugation Experiment and Plasmid Replicon Typing

Conjugation experiments were performed for five *mcr-1* positive *K. pneumoniae*, which also co-harboured *bla*_NDM_. PCR assay showed that *mcr-1* was successfully transferred from four isolates (CR*kp*11, CR*kp*12, CR*kp*13, and CR*kp*17) by conjugation and failed to transfer in one isolate (CR*kp*18). All the transconjugants were phenotypically resistant to colistin but sensitive to imipenem and meropenem. PBRT showed transconjugants of CR*kp*11, CR*kp*12, and CR*kp*13 carried IncFII type plasmid, whilst transconjugants of CR*kp*17 carried an untypeable plasmid. Similarly, PCR assay showed that *bla*_NDM_ was transferred successfully from all five isolates by conjugation, and PBRT results showed that transconjugants of CR*kp*11 and CR*kp*12 carried IncA/C type plasmid whilst transconjugants of CR*kp*13, CR*kp*17 and CR*kp*18 carried IncFIB type plasmid. Phenotypically, all *bla*_NDM_ transconjugants were resistant to imipenem and meropenem but susceptible to colistin.

### S1 PFGE and Southern Hybridisation

S1 PFGE followed by Southern hybridisation showed that *mcr-1* was present both on plasmid and chromosome in three isolates (CR*kp*11, CR*kp*12, andCR*kp*13), whilst one each only on plasmid (CR*kp*17) and chromosome (CR*kp*18). The plasmid size in CR*kp*11, CR*kp*12, and CR*kp*17 was between ~138 and ~210 kb, whilst in CR*kp*13, *mcr-1* was present on a small plasmid between ~33 and ~78 kb (**Figure S1**). The S1 nuclease digested genomic DNA from five *K. pneumoniae* was also probed with digoxigenin labelled *bla*_NDM_, and the results showed that *bla*_NDM_ gene was present both on plasmid (between ~45 and ~400 kb) and chromosome in all five isolates (**Figure S2**).

### GenBank Accession Numbers

The GenBank accession numbers assigned to nucleotide sequences of *mcr-1* were MN652072-MN652090 and for nucleotide sequence of *mgrB* were MW389562–MW389564.

## Discussion

The extensive use of antibiotics for treating infectious diseases has led to the emergence of bacterial antimicrobial resistance. The microbes have benefitted enormously from overuse of antibiotics in clinical practice, also in agricultural and livestock. Emergence and dissemination of transmissible colistin resistance have severely compromised the use of colistin for treatment of infections caused by carbapenem resistant Enterobacteriaceae. In studies reported across the world, *mcr-1* has been predominantly reported in *E. coli* whereas *K. pneumoniae* accounts for less than 5% of the total *mcr* positive isolates to date (Sun et al., 2018; Nang et al., 2019). In contrast to global data, studies from India indicate that colistin resistance is more common in *K. pneumoniae* than in any other bacterial species (Pragasam et al., 2016; Singh et al., 2018; Sodhi et al., 2020). Also, very few studies are available on genomic characterisation of colistin resistant isolates. Hence, we investigated the mechanism of colistin resistance in clinical *K. pneumoniae* isolates and performed genetic characterisation of these isolates to expand our knowledge on colistin resistant *K. pneumoniae*.

*mcr* mediated colistin resistance has been reported across the world, but only few such reports are available from India (Singh et al., 2018; Gogry et al., 2019). We found *mcr-1* mediated colistin resistance in 19 K*. pneumoniae* isolates, whilst insertional inactivation of *mgrB* gene by *IS* elements in three isolates. Insertional inactivation of *mgrB* activates the PhoP/Q two component signalling system that upregulates the *arnBCADTEF* operon which adds 4-amino-4-deoxy-L-arabinose to lipid A resulting in colistin resistance (Cannatelli et al., 2014). Insertional sequences of *IS*1and *IS*5 family are most common *IS* elements responsible for inactivation of *mgrB* gene (Azam et al., 2021). It is noteworthy to mention that we found coexistence of *mcr-1* and *bla*_NDM_ in five *K. pneumoniae* isolates; however studies suggest they are more commonly found in *E. coli* as compared to *K. pneumoniae* (Delgado-Blas et al., 2016; Zheng et al., 2017). Among carbapenemases, *bla*_OXA-48_ was found to be present in all the isolates. In recent years, *bla*_OXA-48_ has increasingly been reported from India; a multi-centric study from India reported the presence of *bla*_OXA-48_ in 80% of the carbapenem resistant isolates (Shankar et al., 2019a). We also observed 39% of our carbapenem resistant isolates were *bla*_OXA-48_ producers (unpublished data). Among MBLs, *bla*_VIM_ was present in 59.1% (13/22) isolates. The unusually high prevalence of *bla*_VIM_ (50% of *bla*_NDM_ positive isolates) was also reported previously from our centre (Rahman et al., 2018). Another study from North India reported *bla*_VIM_ in 18.4% (52/282) of carbapenem resistant isolates (Garg et al., 2019). Aminoglycosides in combination with other antibiotics such as tigecycline are often used for treating infections caused by carbapenem and colistin resistant *K. pneumoniae* (Petrosillo et al., 2019). In the current study, 16S RNA methyltransferase genes were found to be present in seven isolates that also harboured *mcr-1* and *bla*_NDM,_ which indicates towards a grim situation. Among twenty-two patients, twelve (54.5%) succumbed to their disease. We found that the patient death as outcome was attributed to lower respiratory tract infection, blood stream infections, and septic shock caused by MDR *K. pneumoniae*.

The resistance nodulation division acrAB-tolC efflux pumps are reported in diverse members of the Enterobacteriaceae family including *K. pneumoniae*. In *K. pneumoniae* acrAB-tolC efflux pumps have been associated with resistance to *β*-lactams, fluoroquinolones, and tetracycline (Li et al., 2015). Similarly, Multi-Antimicrobial Extrusion mdtK efflux pumps have been also been reported in *K. pneumonia* (Li et al., 2015). In the present study, we detected *acrAB*, *tolc*, and *mdtK* in MDR *K. pneumoniae*. Our results are in concordance with previous studies where authors had shown the presence of drug-resistant genes and efflux pumps in MDR *K. pneumoniae* (Maurya et al., 2019; Ni et al., 2020).

The role of virulence factors in colonisation, invasion, and pathogenicity of *K. pneumoniae* is well known (Paczosa and Mecsas, 2016). Mucoid regulator gene, *rmpA*, is involved in capsule biosynthesis and often associated with hypervirulence was detected in two *K. pneumoniae* isolates (Cheng et al., 2010). The other important virulence factors are siderophores; they are low molecular weight iron scavenging molecules secreted by many GNB that affect the iron homeostasis in host (Page, 2019). In this study, all the colistin resistant *K. pneumoniae* harboured *ent B* and *irp-1* siderophores, which are also known to contribute towards inflammation and bacterial spread during infection (Holden et al., 2016). Adhesin associated genes *fimH*, a type 1 fimbria adhesive subunit and *mrkD*, a type 3 adhesive subunit have been detected in 72.7 and 86.4 isolates respectively. *mrkD* is known to facilitate binding to extracellular matrix which is responsible for bacterial adherence to tissue and indwelling devices such as endotracheal tubes (Paczosa and Mecsas, 2016). Serum resistant outer membrane lipoprotein (*traT*) was detected in 27.3% isolates and reported to play a crucial role in bacterial pathogenesis by blocking the action of membrane attack complex (Miajlovic and Smith, 2014). *K. pneumoniae* is known to produce biofilm which provides a layer of protection by preventing antibiotic penetration and reducing their efficacy. In our study all the isolates were biofilm producer with 50% of them producing strong biofilm, which suggests that MDR *K. pneumoniae* strains are associated with biofilm production.

PFGE is considered gold standard for molecular epidemiology of bacterial strains. PFGE data indicated that the clonal spread of *K. pneumoniae* was not responsible for colistin resistance. The isolates having more than 90% similarity most often were of same ST except in few cases where isolates of same STs clustered separately. Further, the MLST data showed that ST-15 and ST-16 were the most dominant clones followed by ST-231 and ST-147 amongst the *mcr-1* positive *K. pneumoniae*. We found that ST15 *K. pneumoniae* isolate was associated with the presence of *rmpA* gene. Out of four ST-15 isolates, three harboured *bla*_NDM_ and 16S rRNA methyltransferase [*rmtB* (n = 1) and *rmtC* (n = 2)]. All ST15 *K. pneumoniae* were associated with strong biofilm production, whilst the other dominant clone ST16 *K. pneumoniae* was moderate biofilm producers. Previous study from India also supports our data where authors had detected ST-231, ST-14, ST-147, ST-15, ST-16, ST-11, ST-23, and ST-43 in colistin resistant *K. pneumoniae* (Shankar et al., 2019b), whereas global data suggests the presence of heterogeneous STs in *mcr-1* producing *K. pneumoniae*. The diversity in PFGE and ST was also supported by capsular serotyping which predicted eight serotypes based on *wzi* allele sequence. KL10 was the most common capsular serotype detected; in *mcr-1* producing *K. pneumoniae*, KL10 capsulate serotype was associated with *entB* and *irp-1* siderophores along with strong biofilm forming ability.

Conjugation experiments revealed that in four out of five *K. pneumoniae* isolates, *mcr-1* was present on conjugative plasmid. Conjugative plasmids are self-transmissible and are often responsible for rapid spread of resistant traits. Three of the four *mcr-1* transconjugants had IncFII type plasmid, which are conjugative plasmid with low copy number and size ranging between 45 and 200 kb (Rozwandowicz et al., 2018). The role of IncFII type plasmid in dissemination of *mcr-1* is well known (Xavier et al., 2016; Wang et al., 2018). The *mcr-1* harbouring IncFII plasmids were associated with ST15 *K. pneumoniae*. Conjugation experiments in the above five *K. pneumoniae* showed successful transfer of *bla*_NDM_ to recipient *E. coli* J53 that suggests their location on conjugative plasmid. In two transconjugants *bla*_NDM_ was present in IncA/C type plasmid whereas in three transconjugants *bla*_NDM_ was present in IncFIB type plasmid. IncA/C type plasmids are broad host range, low copy number, and frequently found to be responsible for dissemination of *bla*_NDM_. Similarly, previous studies had shown that dissemination of *bla*_NDM_ was linked to transferable IncA/C and IncFIB plasmids (Khan et al., 2017; Sugawara et al., 2019). IncF are considered as epidemic plasmids and linked with the global spread of *K. pneumoniae* ST258 (Rozwandowicz et al., 2018). The presence of multiple plasmids in MDR strains imparts fitness cost; however, it provides bacteria specific traits which help them to survive in stress conditions. S1-PFGE showed that majority of *K. pneumoniae* isolates harboured multiple plasmids. *mcr-1* was present on plasmid of different sizes in these isolates. In three isolates, *mcr-1* was present both on plasmid and chromosome. The chromosomal integration stabilises *mcr-1* and enables it to be vertically transferred without the risk of plasmid loss. Co-existence of transferable *bla*_NDM_ along with *mcr-1* is a major threat to human health by compromising the available treatment options. Previous studies from USA, China, and Vietnam also reported the coexistence of *mcr-1* and *bla*_NDM_ in various members of Enterobacteriaceae and their potential to spread as extensively drug-resistant strains (Mediavilla et al., 2016; Feng et al., 2018; Jin et al., 2018).

In conclusion, *K. pneumoniae* has emerged as the most notorious pathogen among the members of Enterobacteriaceae. They are the reservoirs of diverse resistant traits and virulence genes. Moreover, their biofilm forming ability provides them survival and colonisation advantages. Co-existence of *mcr-1* and *bla*_NDM_ on the transmissible plasmids is a matter of concern as such plasmids possess significant risk of inter- and intra-species dissemination in the environmental and livestock pathogens. Therefore, strict epidemiological surveillance, infection control measures, and antibiotic stewardship are required to curb this menace of colistin resistance from dissemination.

## Data Availability Statement

The datasets presented in this study can be found in online repositories. The names of the repository/repositories and accession number(s) can be found below: https://www.ncbi.nlm.nih.gov/genbank/, MN652072-MN652090 https://www.ncbi.nlm.nih.gov/genbank/, MW389562-MW389564.

## Ethics Statement

The studies involving human participants were reviewed and approved by the Institutional ethics committee of Sanjay Gandhi Postgraduate Institute of Medical Sciences, Lucknow, India [2017-191-PhD-99(B)]. The patients/participants provided their written informed consent to participate in this study.

## Author Contributions

KP conceptualized and supervised the study. SS collected the sample, performed experiments, and drafted the manuscript. AP, MR, and AS performed the experiments and edited the manuscript. SN and CS collected the patient information and provided the demographic data. All authors contributed to the article and approved the submitted version.

## Funding

This study was supported by Science and Engineering Research Board (SERB) (EMR/2015/001804), Government of India.

## Conflict of Interest

The authors declare that the research was conducted in the absence of any commercial or financial relationships that could be construed as a potential conflict of interest.

## Supplementary Material

Supplementary Figure 1 S1-PFGE and Southern blot hybridization of *mcr-1* and *bla*_NDM_ producing *Klebsiella pneumoniae*; **(A, C)** S1 digested DNA analysed by PFGE; **(B, D)** hybridisation of S1-PFGE gel with digoxygenin labelled *mcr-1*gene probe; **(A, C)** Lane M- *Salmonella* Braenderup H9812; **(A–D)** lanes 1—CR*kp*11, 2—CR*kp*12, 3—CR*kp*13, 4—CR*kp*17 and 5—CR*kp*18.

Supplementary Figure 2 S1-PFGE and Southern blot hybridisation of *mcr-1* and *bla*_NDM_ producing *Klebsiella pneumoniae*; **(A)** S1 digested DNA analysed by PFGE, **(B)** hybridisation of S1-PFGE gel with digoxygenin labelled *bla*_NDM_ gene probe; **(A)** lane M—*Salmonella* Braenderup H9812, **(A, B)** lanes 1—CR*kp*11, 2—CR*kp*12, 3—CR*kp*13, 4—CR*kp*17, and 5—CR*kp*18.

## References

[B1] Azam M. Gaind R. Yadav G. Sharma A. Upmanyu K. Jain M.. (2021). Colistin Resistance Among Multiple Sequence Types of Klebsiella Pneumoniae Is Associated With Diverse Resistance Mechanisms: A Report From India. Front. Microbiol. 12, 609840. 10.3389/fmicb.2021.609840 33692764 PMC7937630

[B2] Baron S. Hadjadj L. Rolain J. M. Olaitan A. O. (2016). Molecular Mechanisms of Polymyxin Resistance: Knowns and Unknowns. Int. J. Antimicrob. Agents 48, 583–591. 10.1016/j.ijantimicag.2016.06.023 27524102

[B3] Brisse S. Passet V. Haugaard A. B. Babosan A. Kassis-Chikhani N. Struve C.. (2013). Wzi Gene Sequencing, a Rapid Method for Determination of Capsular Type for Klebsiella Strains. J. Clin. Microbiol. 51, 4073–4078. 10.1128/JCM.01924-13 24088853 PMC3838100

[B4] Cannatelli A. Giani T. D’andrea M. M. Di Pilato V. Arena F. Conte V.. (2014). Mgrb Inactivation Is a Common Mechanism of Colistin Resistance in KPC-Producing Klebsiella Pneumoniae of Clinical Origin. Antimicrob. Agents Chemother. 58, 5696–5703. 10.1128/aac.03110-14 25022583 PMC4187966

[B5] Carattoli A. Bertini A. Villa L. Falbo V. Hopkins K. L. Threlfall E. J. (2005). Identification of Plasmids by PCR-Based Replicon Typing. J. Microbiol. Methods 63, 219–228. 10.1016/j.mimet.2005.03.018 15935499

[B6] Cheng H. Y. Chen Y. S. Wu C. Y. Chang H. Y. Lai Y. C. Peng H. L. (2010). Rmpa Regulation of Capsular Polysaccharide Biosynthesis in Klebsiella Pneumoniae CG43. J. Bacteriol 192, 3144–3158. 10.1128/jb.00031-10 20382770 PMC2901686

[B7] CLSI (2017). “CLSI Supplement M100,” in Performance Standards for Antimicrobial Susceptibility Testing, 27th. Ed. Wayne P. A. (Clinical and Laboratory Standards Institute). 2017.

[B8] Delgado-Blas J. F. Ovejero C. M. Abadia-Patiño L. Gonzalez-Zorn B. (2016). Coexistence of Mcr-1 and blaNDM-1 in Escherichia Coli From Venezuela. Antimicrob. Agents Chemother. 60, 6356–6358. 10.1128/aac.01319-16 27431212 PMC5038285

[B9] Diancourt L. Passet V. Verhoef J. Grimont P. A. Brisse S. (2005). Multilocus Sequence Typing of Klebsiella Pneumoniae Nosocomial Isolates. J. Clin. Microbiol. 43, 4178–4182. 10.1128/jcm.43.8.4178-4182.2005 16081970 PMC1233940

[B10] The European Committee on Antimicrobial Susceptibility Testing (EUCAST). (2017). Breakpoint tables for Interpretation of MICs and Zone Diameters. Version 7.1. Available at: http://www.eucast.org.

[B11] Feng S. Shen C. Chen H. Zheng X. Xia Y. Zhong L. L.. (2018). Co-production of MCR-1 and NDM-5 in Escherichia Coli Isolated From a Colonization Case of Inpatient. Infect. Drug Resist. 11, 1157–1161. 10.2147/idr.s171164 30147343 PMC6098422

[B12] Garg A. Garg J. Kumar S. Bhattacharya A. Agarwal S. Upadhyay G. C. (2019). Molecular Epidemiology & Therapeutic Options of Carbapenem-Resistant Gram-Negative Bacteria. Indian J. Med. Res. 149, 285–289. 10.4103/ijmr.IJMR_36_18 31219096 PMC6563745

[B13] Gogry F. A. Siddiqui M. T. Haq Q. M. R. (2019). Emergence of Mcr-1 Conferred Colistin Resistance Among Bacterial Isolates From Urban Sewage Water in India. Environ. Sci. Pollut. Res. Int. 26, 33715–33717. 10.1007/s11356-019-06561-5 31625114

[B14] Holden V. I. Breen P. Houle S. Dozois C. M. Bachman M. A. (2016). Klebsiella Pneumoniae Siderophores Induce Inflammation, Bacterial Dissemination, and HIF-1α Stabilization During Pneumonia. mBio 7. 10.1128/mBio.01397-16 PMC5021805 27624128

[B15] Jin L. Wang R. Wang X. Wang Q. Zhang Y. Yin Y.. (2018). Emergence of Mcr-1 and Carbapenemase Genes in Hospital Sewage Water in Beijing, China. J. Antimicrob. Chemother. 73, 84–87. 10.1093/jac/dkx355 29040585

[B16] Kado C. I. Liu S. T. (1981). Rapid Procedure for Detection and Isolation of Large and Small Plasmids. J. Bacteriol 145, 1365–1373. 10.1128/jb.145.3.1365-1373.1981 7009583 PMC217141

[B17] Khan A. U. Maryam L. Zarrilli R. (2017). Structure, Genetics and Worldwide Spread of New Delhi Metallo-β-Lactamase (NDM): A Threat to Public Health. BMC Microbiol. 17, 101. 10.1186/s12866-017-1012-8 28449650 PMC5408368

[B18] Laws M. Shaaban A. Rahman K. M. (2019). Antibiotic Resistance Breakers: Current Approaches and Future Directions. FEMS Microbiol. Rev. 43, 490–516. 10.1093/femsre/fuz014 31150547 PMC6736374

[B19] Li X. Z. Plésiat P. Nikaido H. (2015). The Challenge of Efflux-Mediated Antibiotic Resistance in Gram-Negative Bacteria. Clin. Microbiol. Rev. 28, 337–418. 10.1128/cmr.00117-14 25788514 PMC4402952

[B20] Magiorakos A. P. Srinivasan A. Carey R. B. Carmeli Y. Falagas M. E. Giske C. G.. (2012). Multidrug-Resistant, Extensively Drug-Resistant and Pandrug-Resistant Bacteria: An International Expert Proposal for Interim Standard Definitions for Acquired Resistance. Clin. Microbiol. Infect. 18, 268–281. 10.1111/j.1469-0691.2011.03570.x 21793988

[B21] Maurya N. Jangra M. Tambat R. Nandanwar H. (2019). Alliance of Efflux Pumps With β-Lactamases in Multidrug-Resistant Klebsiella Pneumoniae Isolates. Microb. Drug Resist. 25, 1155–1163. 10.1089/mdr.2018.0414 31613200 PMC6807647

[B22] Mediavilla J. R. Patrawalla A. Chen L. Chavda K. D. Mathema B. Vinnard C.. (2016). Colistin- and Carbapenem-Resistant Escherichia Coli Harboring Mcr-1 and Blandm-5, Causing a Complicated Urinary Tract Infection in a Patient From the United States. mBio 7. 10.1128/mBio.01191-16 PMC4999550 27578755

[B23] Miajlovic H. Smith S. G. (2014). Bacterial Self-Defence: How Escherichia Coli Evades Serum Killing. FEMS Microbiol. Lett. 354, 1–9. 10.1111/1574-6968.12419 24617921

[B24] Nang S. C. Li J. Velkov T. (2019). The Rise and Spread of Mcr Plasmid-Mediated Polymyxin Resistance. Crit. Rev. Microbiol. 45, 131–161. 10.1080/1040841x.2018.1492902 31122100 PMC6625916

[B25] Navon-Venezia S. Kondratyeva K. Carattoli A. (2017). Klebsiella Pneumoniae: A Major Worldwide Source and Shuttle for Antibiotic Resistance. FEMS Microbiol. Rev. 41, 252–275. 10.1093/femsre/fux013 28521338

[B26] Ni R. T. Onishi M. Mizusawa M. Kitagawa R. Kishino T. Matsubara F.. (2020). The Role of RND-Type Efflux Pumps in Multidrug-Resistant Mutants of Klebsiella Pneumoniae. Sci. Rep. 10, 10876. 10.1038/s41598-020-67820-x 32616840 PMC7331594

[B27] O’toole G. A. Kolter R. (1998). Initiation of Biofilm Formation in Pseudomonas Fluorescens WCS365 Proceeds *Via* Multiple, Convergent Signalling Pathways: A Genetic Analysis. Mol. Microbiol. 28, 449–461. 10.1046/j.1365-2958.1998.00797.x 9632250

[B28] Paczosa M. K. Mecsas J. (2016). Klebsiella Pneumoniae: Going on the Offense With a Strong Defense. Microbiol. Mol. Biol. Rev. 80, 629–661. 10.1128/mmbr.00078-15 27307579 PMC4981674

[B29] Page M. G. P. (2019). The Role of Iron and Siderophores in Infection, and the Development of Siderophore Antibiotics. Clin. Infect. Dis. 69, S529–s537. 10.1093/cid/ciz825 31724044 PMC6853763

[B30] Petrosillo N. Taglietti F. Granata G. (2019). Treatment Options for Colistin Resistant Klebsiella Pneumoniae: Present and Future. J. Clin. Med. 8. 10.3390/jcm8070934 PMC6678465 31261755

[B31] Pragasam A. K. Shankar C. Veeraraghavan B. Biswas I. Nabarro L. E. Inbanathan F. Y.. (2016). Molecular Mechanisms of Colistin Resistance in Klebsiella Pneumoniae Causing Bacteremia From India-A First Report. Front. Microbiol. 7, 2135. 10.3389/fmicb.2016.02135 28119670 PMC5220082

[B32] Qin S. Fu Y. Zhang Q. Qi H. Wen J. G. Xu H.. (2014). High Incidence and Endemic Spread of NDM-1-Positive Enterobacteriaceae in Henan Province, China. Antimicrob. Agents Chemother. 58, 4275–4282. 10.1128/aac.02813-13 24777095 PMC4136005

[B33] Rahman M. Prasad K. N. Gupta S. Singh S. Singh A. Pathak A.. (2018). Prevalence and Molecular Characterization of New Delhi Metallo-Beta-Lactamases in Multidrug-Resistant Pseudomonas Aeruginosa and Acinetobacter Baumannii From India. Microb. Drug Resist. 24, 792–798. 10.1089/mdr.2017.0078 29058515

[B34] Rozwandowicz M. Brouwer M. S. M. Fischer J. Wagenaar J. A. Gonzalez-Zorn B. Guerra B.. (2018). Plasmids Carrying Antimicrobial Resistance Genes in Enterobacteriaceae. J. Antimicrob. Chemother. 73, 1121–1137. 10.1093/jac/dkx488 29370371

[B35] Shankar C. Mathur P. Venkatesan M. Pragasam A. K. Anandan S. Khurana S.. (2019a). Rapidly Disseminating Bla(OXA-232) Carrying Klebsiella Pneumoniae Belonging to ST231 in India: Multiple and Varied Mobile Genetic Elements. BMC Microbiol. 19, 137. 10.1186/s12866-019-1513-8 31234800 PMC6591861

[B36] Shankar C. Venkatesan M. Rajan R. Mani D. Lal B. Anandan S.. (2019b). Molecular Characterization of Colistin-Resistant Klebsiella Pneumoniae & Its Clonal Relationship Among Indian Isolates. Indian J. Med. Res. 149, 199–207. 10.4103/ijmr.IJMR_2087_17 31219084 PMC6563726

[B37] Singh S. Pathak A. Kumar A. Rahman M. Singh A. Gonzalez-Zorn B.. (2018). Emergence of Chromosome-Borne Colistin Resistance Gene Mcr-1 in Clinical Isolates of Klebsiella Pneumoniae From India. Antimicrob. Agents Chemother. 62. 10.1128/aac.01885-17 PMC5786762 29133565

[B38] Sodhi K. Mittal V. Arya M. Kumar M. Phillips A. Kajla B. (2020). Pattern of Colistin Resistance in Klebsiella Isolates in an Intensive Care Unit of a Tertiary Care Hospital in India. J. Infect. Public Health 13, 1018–1021. 10.1016/j.jiph.2019.10.013 31818712

[B39] Stepanovic S. Vukovic D. Dakic I. Savic B. Svabic-Vlahovic M. (2000). A Modified Microtiter-Plate Test for Quantification of Staphylococcal Biofilm Formation. J. Microbiol. Methods 40, 175–179. 10.1016/s0167-7012(00)00122-6 10699673

[B40] Sugawara Y. Akeda Y. Hagiya H. Sakamoto N. Takeuchi D. Shanmugakani R. K.. (2019). Spreading Patterns of NDM-Producing Enterobacteriaceae in Clinical and Environmental Settings in Yangon, Myanmar. Antimicrob. Agents Chemother. 63 (3), e01924–18. 10.1128/aac.01924-18 30530602 PMC6395922

[B41] Sun J. Zhang H. Liu Y. H. Feng Y. (2018). Towards Understanding MCR-Like Colistin Resistance. Trends Microbiol. 26, 794–808. 10.1016/j.tim.2018.02.006 29525421

[B42] Wang C. Feng Y. Liu L. Wei L. Kang M. Zong Z. (2020). Identification of Novel Mobile Colistin Resistance Gene Mcr-10. Emerg. Microbes Infect. 9, 508–516. 10.1080/22221751.2020.1732231 32116151 PMC7067168

[B43] Wang R. Van Dorp L. Shaw L. P. Bradley P. Wang Q. Wang X.. (2018). The Global Distribution and Spread of the Mobilized Colistin Resistance Gene Mcr-1. Nat. Commun. 9, 1179. 10.1038/s41467-018-03205-z 29563494 PMC5862964

[B44] WHO (2017). WHO Publishes List of Bacteria for Which New Antibiotics are Urgently Needed (Geneva, Switzerland: World Health Organization).

[B45] Xavier B. B. Lammens C. Butaye P. Goossens H. Malhotra-Kumar S. (2016). Complete Sequence of an IncFII Plasmid Harbouring the Colistin Resistance Gene Mcr-1 Isolated From Belgian Pig Farms. J. Antimicrob. Chemother. 71, 2342–2344. 10.1093/jac/dkw191 27261261

[B46] Zheng B. Yu X. Xu H. Guo L. Zhang J. Huang C.. (2017). Complete Genome Sequencing and Genomic Characterization of Two Escherichia Coli Strains Co-Producing MCR-1 and NDM-1 From Bloodstream Infection. Sci. Rep. 7, 17885. 10.1038/s41598-017-18273-2 29263349 PMC5738369

