# Supplementary figures and images for: Genetic Characterisation of Colistin Resistant Klebsiella pneumoniae Clinical Isolates From North India
Source: Front Cell Infect Microbiol. 2021 Jun 21;11:666030. doi: 10.3389/fcimb.2021.666030 (PMC8256276; doi:10.3389/fcimb.2021.666030)

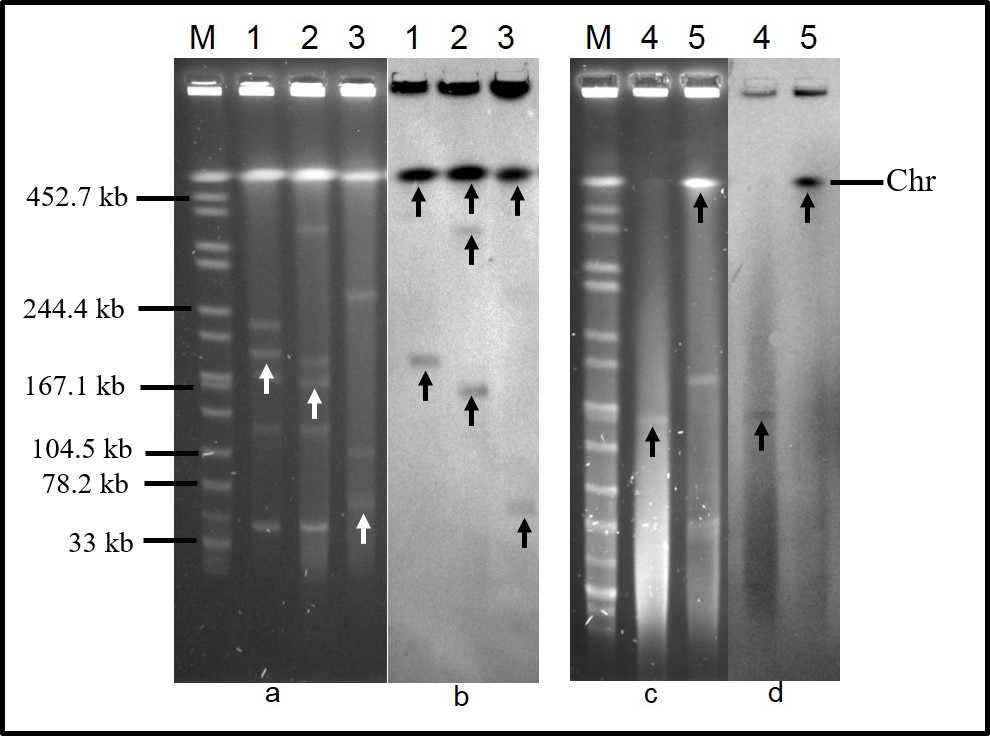

Supplement: Supplementary Figure 1 — S1-PFGE and Southern blot hybridization of mcr-1 and bla NDM producing Klebsiella pneumoniae; (A, C) S1 digested DNA analysed by PFGE; (B, D) hybridisation of S1-PFGE gel with digoxygenin labelled mcr-1gene probe; (A, C) Lane M- Salmonella Braenderup H9812; (A–D) lanes 1—CRkp11, 2—CRkp12, 3—CRkp13, 4—CRkp17 and 5—CRkp18. [file Image_1.jpeg]

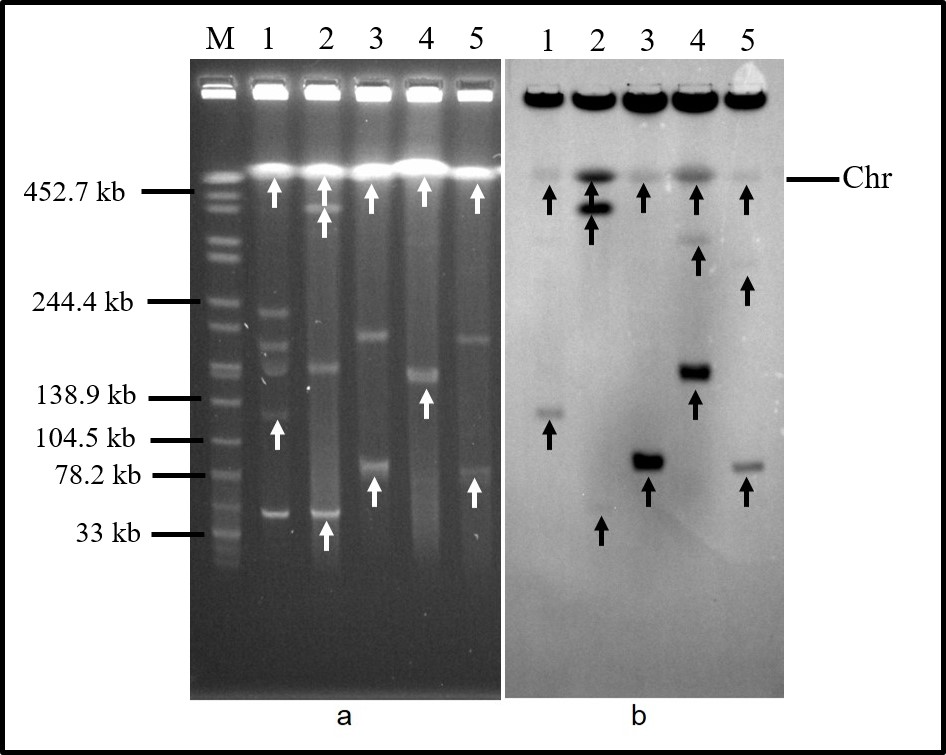

Supplement: Supplementary Figure 2 — S1-PFGE and Southern blot hybridisation of mcr-1 and bla NDM producing Klebsiella pneumoniae; (A) S1 digested DNA analysed by PFGE, (B) hybridisation of S1-PFGE gel with digoxygenin labelled bla NDM gene probe; (A) lane M—Salmonella Braenderup H9812, (A, B) lanes 1—CRkp11, 2—CRkp12, 3—CRkp13, 4—CRkp17, and 5—CRkp18. [file Image_2.jpeg]
