# Supplementary material for: Genetic Characterisation of Colistin Resistant Klebsiella pneumoniae Clinical Isolates From North India
Source: Front Cell Infect Microbiol. 2021 Jun 21;11:666030. doi: 10.3389/fcimb.2021.666030 (PMC8256276; doi:10.3389/fcimb.2021.666030)
Supplement: Supplementary file 3 [file Table_1.docx]

Table S1. List of primers used in the study

| Gene | Forward primer (5’→3’) | Reverse primer (5’→3’) | Amplicon size  ( base pairs) | References |
| --- | --- | --- | --- | --- |
| *mcr-1* | CGGTCAGTCCGTTTGTTC | CTTGGTCGGTCTGTAGGG | 309 | Haeili et al., 2017 |
| *mcr-2* | TGTTGCTTGTGCCGATTGGA | AGATGGTATTGTTGGTTGCTG | 567 | Haeili et al., 2017 |
| *mcr-3* | TTGGCACTGTATTTTGCATTT | TTAACGAAATTGGCTGGAACA | 542 | Haeili et al., 2017 |
| *mcr-4* | ATTGGGATAGTCGCCTTTTT | TTACAGCCAGAATCATTATCA | 487 | Haeili et al., 2017 |
| *mcr-5* | ATGCGGTTGTCTGCATTTATC | TCATTGTGGTTGTCCTTTTCTG | 1644 | ECDC, 2019. |
| *mcr-6* | GTCCGGTCAATCCCTATCTGT | ATCACGGGATTGACATAGCTAC | 556 | ECDC, 2019. |
| *mcr-7* | TGCTCAAGCCCTTCTTTTCGT | TTCATCTGCGCCACCTCGT | 892 | ECDC, 2019. |
| *mcr-8* | AACCGCCAGAGCACAGAATT | TTCCCCCAGCGATTCTCCAT | 667 | ECDC, 2019. |
| *mgrB* | ACCACCTCAAAGAGAAGGCGTT | GGCGTGATTTTGACACGAACAC | 347 | Haeili et al., 2017 |
| *PhoP* | GAGCGTCAGACTACTATCGA | GTTTTCCCATCTCGCCAGCA | 912 | Haeili et al., 2017 |
| *PhoQ* | CCACAGGACGTCATCACCA | GCAGGTGTCTGACAGGGATT | 1594 | Haeili et al., 2017 |
| *PmrA* | CGCAGGATAATCTGTTCTCCA | GGTCCAGGTTTCAGTTGCAA | 808 | Haeili et al., 2017 |
| *PmrB* | ACCTACGCGAAAAGATTGGC | GATGAGGATAGCGCCCATGC | 1274 | Haeili et al., 2017 |
| *bla*_KPC_ | TGTCACTGTATCGCCGTC | GTCAGTGCTCTACAGAAAACC | 1011 | Yigit et al., 2001 |
| *bla*_OXA-48_ | TATATTGCATTAAGCAAGGG | CACACAAATACGCGCTAACC | 848 | Potron et al., 2011 |
| *bla*_NDM_ | CACCT CATGTTTGAATTCGCC | CTCTGTCACATCGAAATCGC | 984 | Bonnin et al., 2012 |
| *bla*_VIM_ | GATGGTGTTTGGTCGCAT | CGAATGCGCAGCACCAG | 390 | Ellington et al., 2007 |
| *bla*_IMP_ | GGAATAGAGTGGCTTAAYTCTC | CCAAACYACTASGTTATCT | 188 | Ellington et al., 2007 |
| *bla*_SHV_ | GGGTTATTCTTATTTGTCGCT | TAGCGTTGCCAGTGCTCG | 929 | Bora et al., 2014 |
| *bla*_TEM_ | AAAATTCTTGAACG | TTACCAAATGCTTAATCA | 1080 | Bora et al., 2014 |
| *bla*_CTX-M_ | TTTGCGATGTGCAGTACCAGTAA | CGATATCGTTGGTGGTGCCATA | 544 | Bora et al., 2014 |
| *armA* | CAAATGGATAAGAATGATGTT | TTATTTCTGAAATCCACT | 774 | Hidalgo et al., 2013 |
| *rmtA* | ATGAGCTTTGACGATGCCCTA | TCACTTATTCCTTTTTATCATG | 756 | Hidalgo et al., 2013 |
| *rmtB* | ATGAACATCAACGATGCCCT | CCTTCTGATTGGCTTATCCA | 769 | Hidalgo et al., 2013 |
| *rmtC* | CGAAGAAGTAACAGCCAAAG | ATCCCAACATCTCTCCCACT | 711 | Hidalgo et al., 2013 |
| *rmtD* | CGGCACGCGATTGGGAAGC | CGGAAACGATGCGACGAT | 401 | Hidalgo et al., 2013 |
| *rmtE* | ATGAATATTGATGAAATGGTTGC | TGATTGATTTCCTCCGTTTTTG | 818 | Hidalgo et al., 2013 |
| *rmtF* | GCGATACAGAAAACCGAAGG | ACCAGTCGGCATAGTGCTTT | 589 | Hidalgo et al., 2013 |

**References**

Haeili, M., Javani, A., Moradi, J., Jafari, Z., Feizabadi, M.M., and Babaei, E. (2017). MgrB Alterations Mediate Colistin Resistance in Klebsiella pneumoniae Isolates from Iran. *Front Microbiol* 8**,** 2470.doi 10.3389/fmicb.2017.02470.

European Centre for Disease Prevention and Control (ECDC). Laboratory manual for carbapenem and colistin resistance detection and characterisation for the survey of carbapenem- and/or colistin-resistant Enterobacteriaceae – version 2.0. ECDC; 2019.

Yigit, H., Queenan, A.M., Anderson, G.J., Domenech-Sanchez, A., Biddle, J.W., Steward, C.D., Alberti, S., Bush, K., and Tenover, F.C. (2001). Novel carbapenem-hydrolyzing beta-lactamase, KPC-1, from a carbapenem-resistant strain of Klebsiella pneumoniae. *Antimicrob Agents Chemother* 45**,** 1151-1161.doi 10.1128/aac.45.4.1151-1161.2001.

Potron, A., Nordmann, P., Lafeuille, E., Al Maskari, Z., Al Rashdi, F., and Poirel, L. (2011). Characterization of OXA-181, a carbapenem-hydrolyzing class D beta-lactamase from Klebsiella pneumoniae. *Antimicrob Agents Chemother* 55**,** 4896-4899.doi 10.1128/aac.00481-11.

Bonnin, R.A., Naas, T., Poirel, L., and Nordmann, P. (2012). Phenotypic, biochemical, and molecular techniques for detection of metallo-β-lactamase NDM in Acinetobacter baumannii. *J Clin Microbiol* 50**,** 1419-1421.doi 10.1128/jcm.06276-11.

Ellington, M.J., Kistler, J., Livermore, D.M., and Woodford, N. (2007). Multiplex PCR for rapid detection of genes encoding acquired metallo-beta-lactamases. *J Antimicrob Chemother* 59**,** 321-322.doi 10.1093/jac/dkl481.

Bora, A., Hazarika, N.K., Shukla, S.K., Prasad, K.N., Sarma, J.B., and Ahmed, G. (2014). Prevalence of blaTEM , blaSHV and blaCTX-M genes in clinical isolates of Escherichia coli and Klebsiella pneumoniae from Northeast India. *Indian J Pathol Microbiol* 57**,** 249-254.doi 10.4103/0377-4929.134698.

Hidalgo, L., Hopkins, K.L., Gutierrez, B., Ovejero, C.M., Shukla, S., Douthwaite, S., Prasad, K.N., Woodford, N., and Gonzalez-Zorn, B. (2013). Association of the novel aminoglycoside resistance determinant RmtF with NDM carbapenemase in Enterobacteriaceae isolated in India and the UK. *J Antimicrob Chemother* 68**,** 1543-1550.doi 10.1093/jac/dkt078.
